# Green synthesis of selenium nanoparticles mediated from *Ceropegia bulbosa* Roxb extract and its cytotoxicity, antimicrobial, mosquitocidal and photocatalytic activities

**DOI:** 10.1038/s41598-020-80327-9

**Published:** 2021-01-13

**Authors:** Vetrivel Cittrarasu, Durairaj Kaliannan, Kalaimurugan Dharman, Viji Maluventhen, Murugesh Easwaran, Wen Chao Liu, Balamuralikrishnan Balasubramanian, Maruthupandian Arumugam

**Affiliations:** 1 Ethnopharmacology and Algal Biotechnology Division, Department of Botany, School of Life Sciences, Periyar University, Salem, Tamil Nadu 636011 India; 2 Department of Environmental Science, School of Life Sciences, Periyar University, Salem, Tamil Nadu 636011 India; 3 Zoonosis Research Center, Department of Infection Biology, School of Medicine, Wonkwang University, Iksan, 54538 South Korea; 4 Department of Botany, Thiagarajar College, Madurai, Tamil Nadu 625 099 India; 5 Nutritional Improvement of Crops, International Centre for Genetic Engineering and Biotechnology, New Delhi, 110067 India; 6 Department of Animal Science, College of Coastal Agricultural Sciences, Guangdong Ocean University, Zhanjiang, 524088 P. R. China; 7 Department of Food Science and Biotechnology, College of Life Sciences, Sejong University, Seoul, 05006 South Korea

**Keywords:** Biological techniques, Cancer, Computational biology and bioinformatics, Microbiology, Molecular biology

## Abstract

The present study is to design an eco-friendly mode to rapidly synthesize selenium nanoparticles (SeNPs) through *Ceropegia bulbosa* tuber’s aqueous extracts and confirming SeNPs synthesis by UV–Vis spectroscopy, FT-IR, XRD, FE-SEM-EDS mapping, HR-TEM, DLS and zeta potential analysis. In addition, to assess the anti-cancer efficacy of the SeNPs against the cultured MDA-MB-231, as studies have shown SeNPs biosynthesis downregulates the cancer cells when compared to normal HBL100 cell lines. The study observed the IC_50_ value of SeNPs against MDA-MB-231 cells was 34 µg/mL for 48 h. Furthermore, the SeNPs promotes growth inhibitory effects of certain clinical pathogens such as *Bacillus subtilis* and *Escherichia coli*. Apart, from this the SeNPs has shown larvicidal activity after 24 h exposure in *Aedes albopitus* mosquito’s larvae with a maximum of 250 g/mL mortality concentration. This is confirmed by the histopathology results taken at the 4th larval stage. The histopathological studies revealed intense deterioration in the hindgut, epithelial cells, mid gut and cortex region of the larvae. Finally, tried to investigate the photocatalytic activity of SeNPs against the toxic dye, methylene blue using halogen lamp and obtained 96% degradation results. Withal computational study SeNPs was shown to exhibit consistent stability towards breast cancer protein BRCA2. Overall, our findings suggest SeNPs as a potent disruptive agent for MDA-MB-231 cells, few pathogens, mosquito larvae and boosts the photocatalytic dye degradation.

## Introduction

In recent decades, metallic nanoparticles (Silver, Gold, Selenium, Zinc oxide, and Copper oxide) have been rapidly synthesized through biological or green chemistry methods and these nanoparticles are produced in pure, non-toxic and environmental friendly mode by utilizing high-energy renewable materials to promotes the performance and safety in the nanoparticle developing processes^1,2^. Developing plant-mediated nanoparticles is relatively faster as there is no need for maintaining specific conditions in media and culture as it is required for other biological organisms. Additionally, plant extracts consists of cofactors enzymes, flavonoids, proteins and terpenoids to serve as reducing and capping agents^3^. The fabricated nanoparticles are already in practice in diverse fields like solar energy conversion, catalysis, water treatment, medicine and may aid in dealing with technological and environmental challenges^4^. Habitually, green route nanoparticles possess high catalytic ability due to high surface area and the ability to increase reactivity by producing reactive oxygen species, which triggers higher toxicity in bacteria’s and carcinomas^5,6^.

Global impact of disease like cancer and fever is high due to different living habits e.g. modern industrialization for instance doesn’t affect the humans alone, it hinders the path of the entire ecosystem. However, peoples expect to solve such problems quickly with their technologies advances. Mosquito’s serve as an important vector for transmitting several diseases significantly causing fevers are dengue, zika, and chikungunya fever. Presently, mosquito’s act a massive spreader of urban diseases in rural India. To deal with this cataclysm researchers recommend advanced green nanotechnology technics to acquire solutions for vector disease^7^. Selenium is one of the critical trace elements required for normal functioning in the living cells of mammals and higher animals^8–10^. Nowadays, selenium nanoparticles (SeNPs) are accepted by many enthusiastic researchers and recommended for use in various scientific disciplines due to their less toxicity and high stability^11^. Also, it can be synthesized by different approaches like physical, chemical, and biological. According to Wadhwani et al.^12^ statement, biologically mediated SeNPs are safer, environmental friendly and economically viable when compared with other approaches (chemically and physical). At the same time, reports are available on biological approach to produce SeNPs via plants part such as dried leaf, seed, flowers and bark^13,14^. The *Ceropegia bulbosa* Roxb. var. *bulbosa* have been endangered plant from *Asclepia daceae* family with wide therapeutically properties. The *C. bulbosa* leaves are edible and considered as digestive tonic for wellness^15^. The genus *Ceropegia* as a whole is under threat due to destructive collection and habitat degradation. The *C. bulbosa* is used to improve the defense mechanism in the body, and their tubers are used to treat stones in kidney, urinary tract infections, and mere eating has shown to enhance fertility and viability in women^16^.

Therefore, the focal aim of this present investigation is to rapidly synthesize SeNPs from *C. bulbosa* tuber aqueous extract and to determine its potential ability in varied turmoils (i) cytotoxic effects against human breast malignance cells (MDA-MB-231); (ii) antibacterial activity against human clinical pathogens and their morphological analysis; (iii) toxicity bioassay against *Aedes albopictus* mosquito larvae along with the histopathological analysis; (iv) photocatalytic activity using the methylene blue (MB) dye.

## Results and discussion

### Synthesis and characterization of Se-NPs

**Figure 1:**
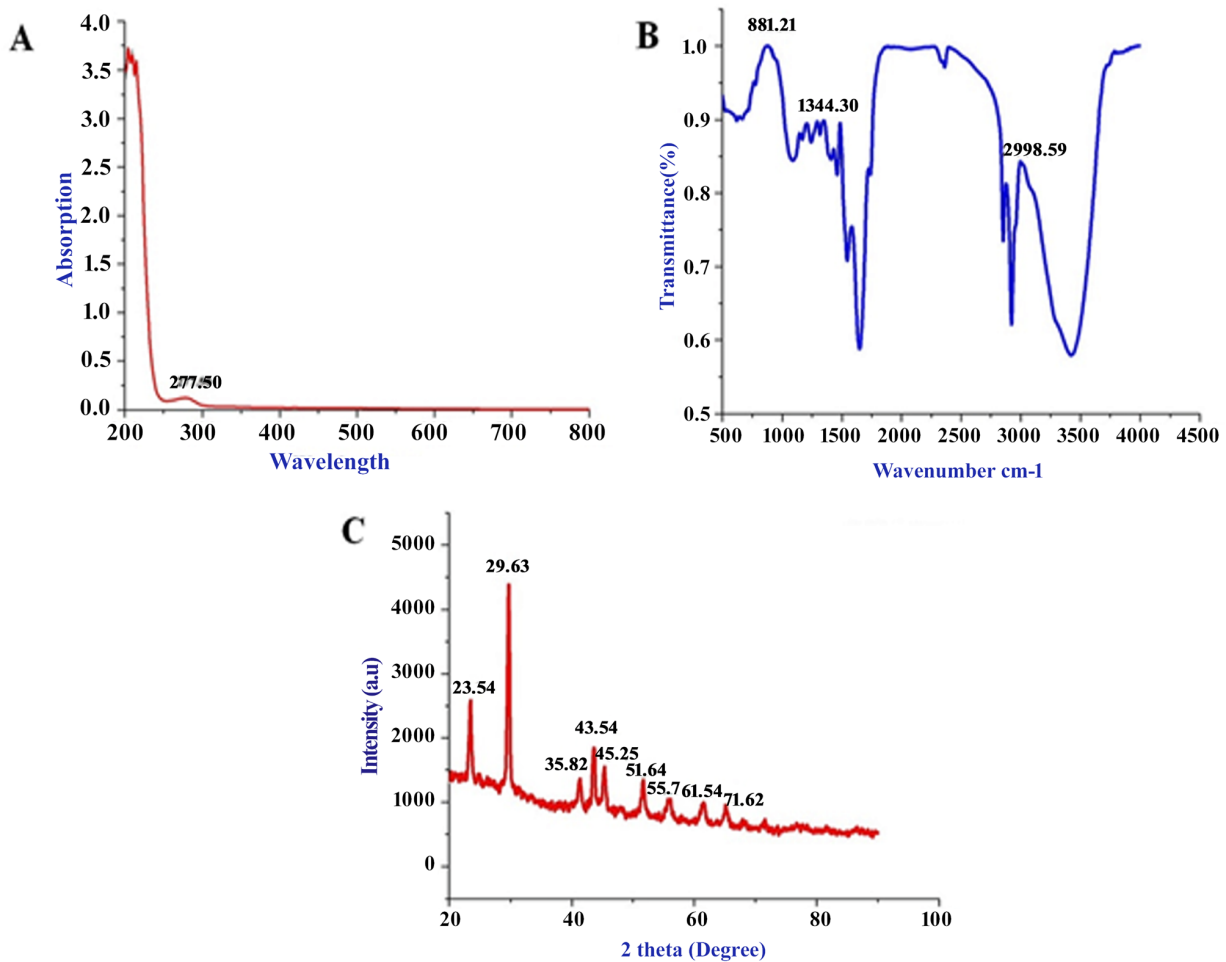
Rapid synthesized SeNPs (**A**) UV–visible absorbance spectrum analysis, (**B**) FT-IR analysis and (**C**) XRD analysis.

The SeNPs is successfully synthesized from *C. bulbosa* tuber aqueous extract rapidly. The changes of color yellow to ruby red indicated the synthesis of SeNPs and is preliminarily substantiated by UV–Vis spectrum high peak absorption spectrum at 277.5 nm (Fig. 1A); and the formation of such peak occurs due to the Surface Plasmon Resonance (SPR) of SeNPs. The result of wide SPR peak strongly reveals the polydispersity of the SeNPs. Similarly, few studies has reported SeNPs synthesis using different reductant agents^17–19^. The Fourier Transform Infrared spectrometer (FTIR) spectrum rapidly synthesize SeNPs as presented in Fig. 1B. A broad vibration peak at 3430 cm^−1^ corresponds to O–H stretch of alcohols and phenols groups. Absorption peak at 2977 and 2880 cm^−1^ represents the C–H stretch of alkynes groups. The small band at 2320 cm^−1^ corresponds nitro compounds (N–O asymmetric stretch) present in the compound. The strong band at 1570 and 1480 cm^−1^ relates to the aromatics and alkanes ring (C–C and C–H stretching). The small vibrational peaks 1376 to 1250 cm^−1^ corresponds to the bending C–H, C–N, O–H, C–X and C–N–C stretches attributed to alkanes, amines and carboxylic groups. However, these functional groups are confirms the involvement of different reducing and stabilizing agents in the synthesis of SeNPs^20^.

**Figure 2:**
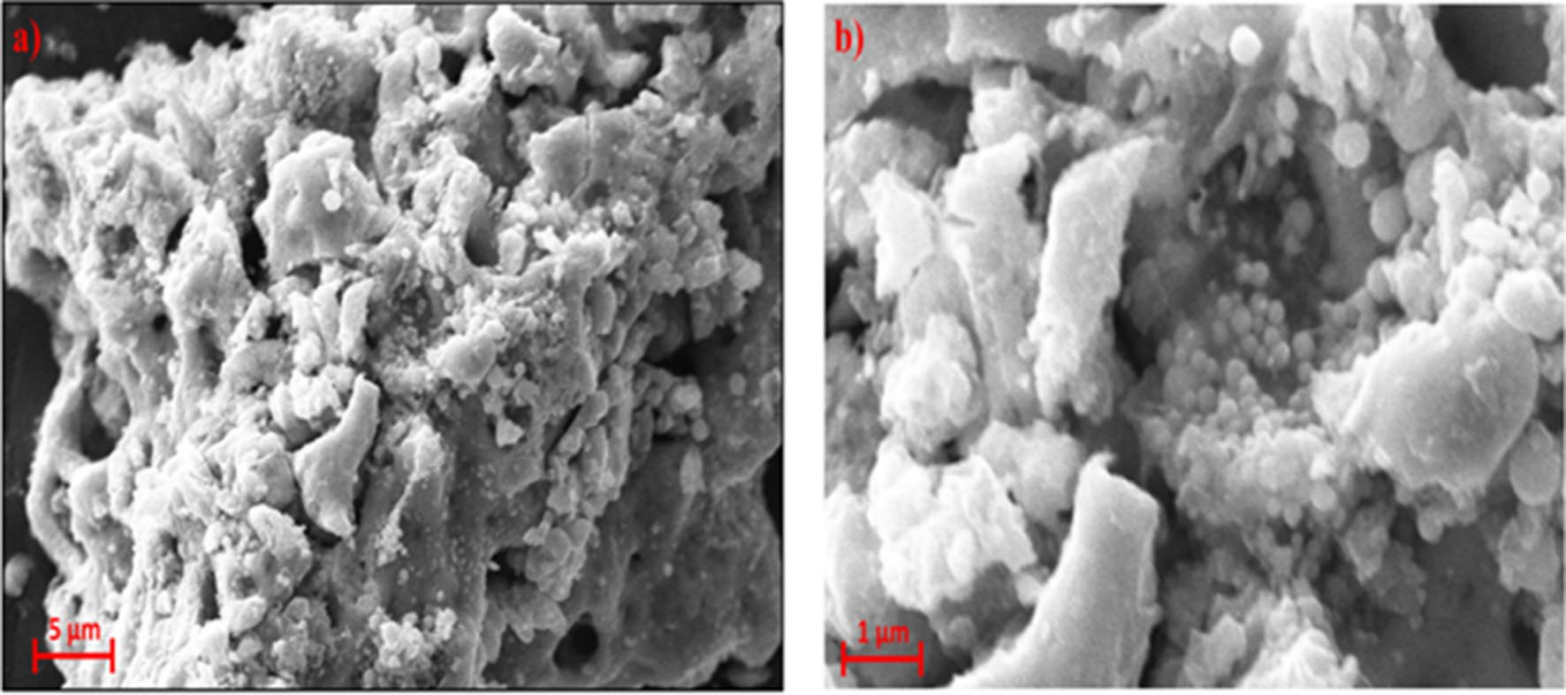
Different magnification of (**a**,**b**) rapid synthesized SeNPs FE-SEM images (**a**) 5 µm, (**b**) 1 µm.

**Figure 3:**
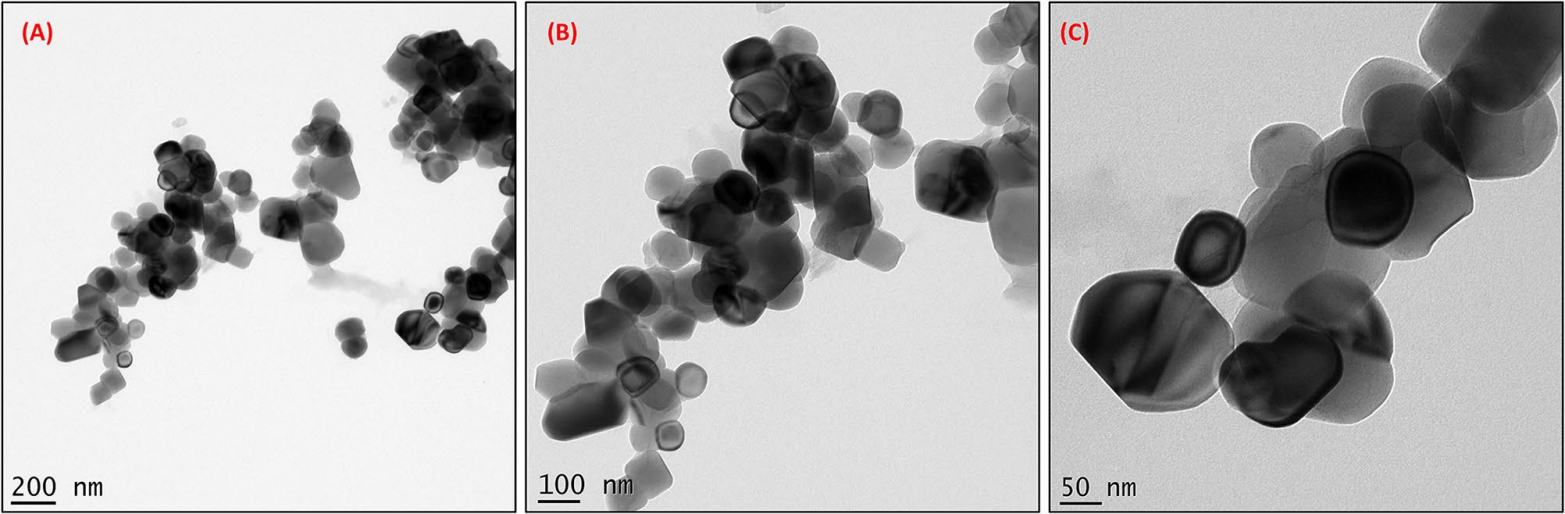
Different magnification of rapid synthesized SeNPs HR-TEM images (**A**) 200 nm, (**B**) 100 nm, (**C**) 50 nm.

**Figure 4:**
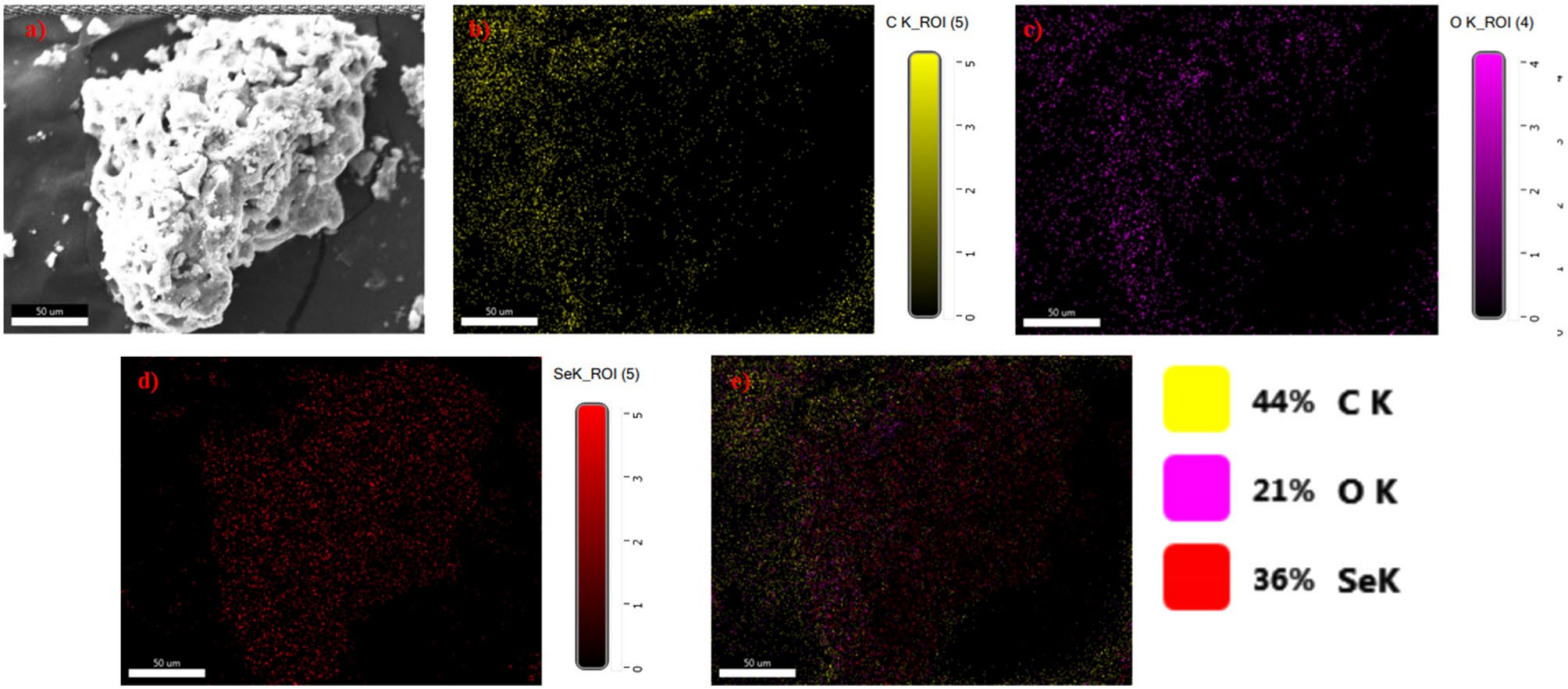
Rapid synthesized SeNPs FE-SEM elemental mapping analysis (**a**) original FE-SEM image, (**b**) carbon element, (**c**) oxygen element, (**d**) Selenium element and (**e**) all element combines with original FE-SEM image.

**Figure 5:**
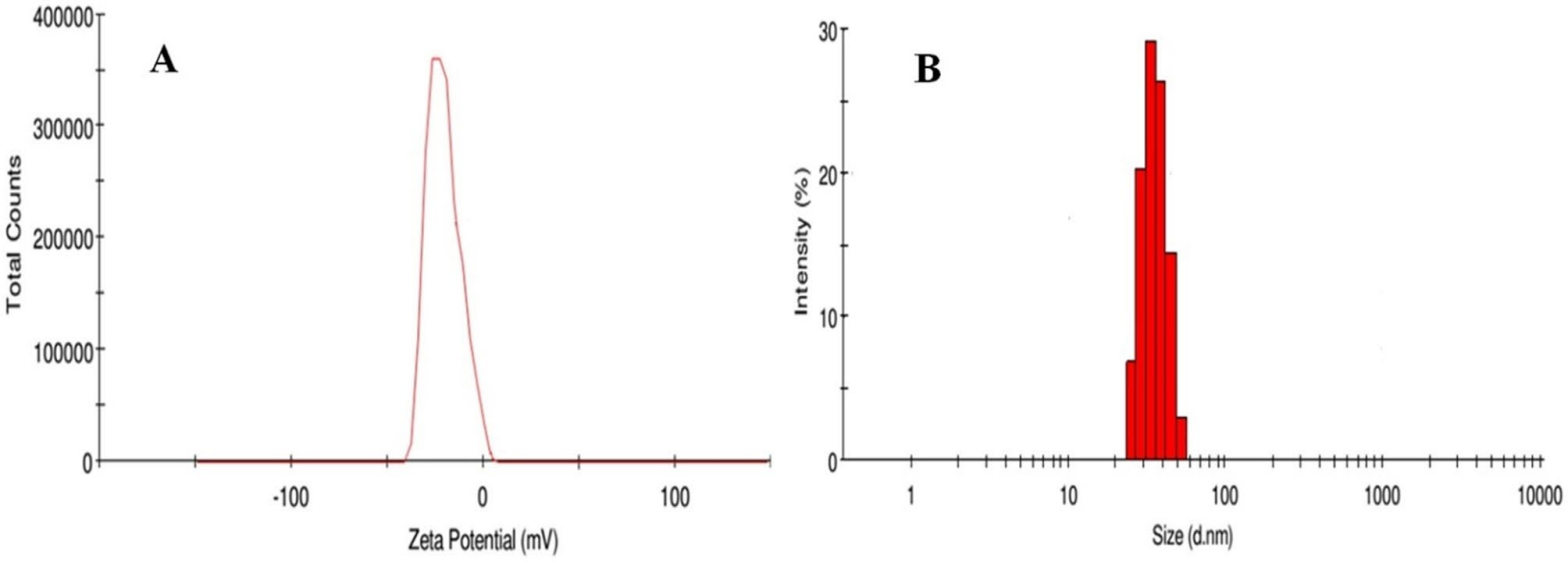
Rapid synthesized SeNPs (**A**) Zeta potential analysis (**B**) DLS analysis.

Rapid synthesis of SeNPs was sturdily confirmed by X-ray diffractometer (XRD) analysis as appeared in Fig. 1C. The SeNPs X-ray plans (100), (101), (110), (102), (111), (201), (003), (202) and (210) were matched at diffraction peaks at 2Ɵ of 23.56°, 29.68°, 43.56°, 45.28°, 51.64°, 55.7°, 61.54°, 71,62° and 78.44°. These reflection plans shows the diffraction peaks were well synchronized and were confirmed with JCPDS (Joint Committee on Powder Diffraction Standards) file no. 06-0362 files, evidently representing the crystalline nature of SeNPs which is in agreement with the works of Alam et al.^14^, showing SeNPs obtained from *Withania somnifera* leaf aqueous extract were crystalline in nature. The SeNPs morphology was confirmed by Field Emission Scanning Electron Microscope (FE-SEM) and High-Resolution Transmission Electron Microscope (HR-TEM) analysis. The FE-SEM (Fig. 2a,b) and HR-TEM (Fig. 3a–c) images clearly reveals the mono scattering and uniform spherical morphology of the particles. Further the elements were confirmed by SEM–EDS mapping analysis. The mapping (Fig. 4a–e) analysis detected the presence of higher weight selenium elements percentage in nanoparticle. These results were sturdily confirmed for the rapid synthesis of SeNPs. Following the zeta potential (ZP) and dynamic light scattering (DLS) results the surface charge and size of SeNPs were assumed as it is an important micronutrient or catalyst of the biological and environmental systems. Additionally, average particle size was re-confirmed by Image J analysis based on the Fig. 3 (Supplementary Fig. S1, Supplementary Table 4). The ZP results of SeNPs are presented as (Fig. 5A) negative charge value of − 17.8 mV. This negatively charged SeNPs can be an potent environment pollutants degradation agent^4^. In addition, the SeNPs DLS results as shown in Fig. 5B shows an average size of 55.9 nm with a polydispersity index of 0.03.

### In vitro cytotoxicity

**Figure 6:**
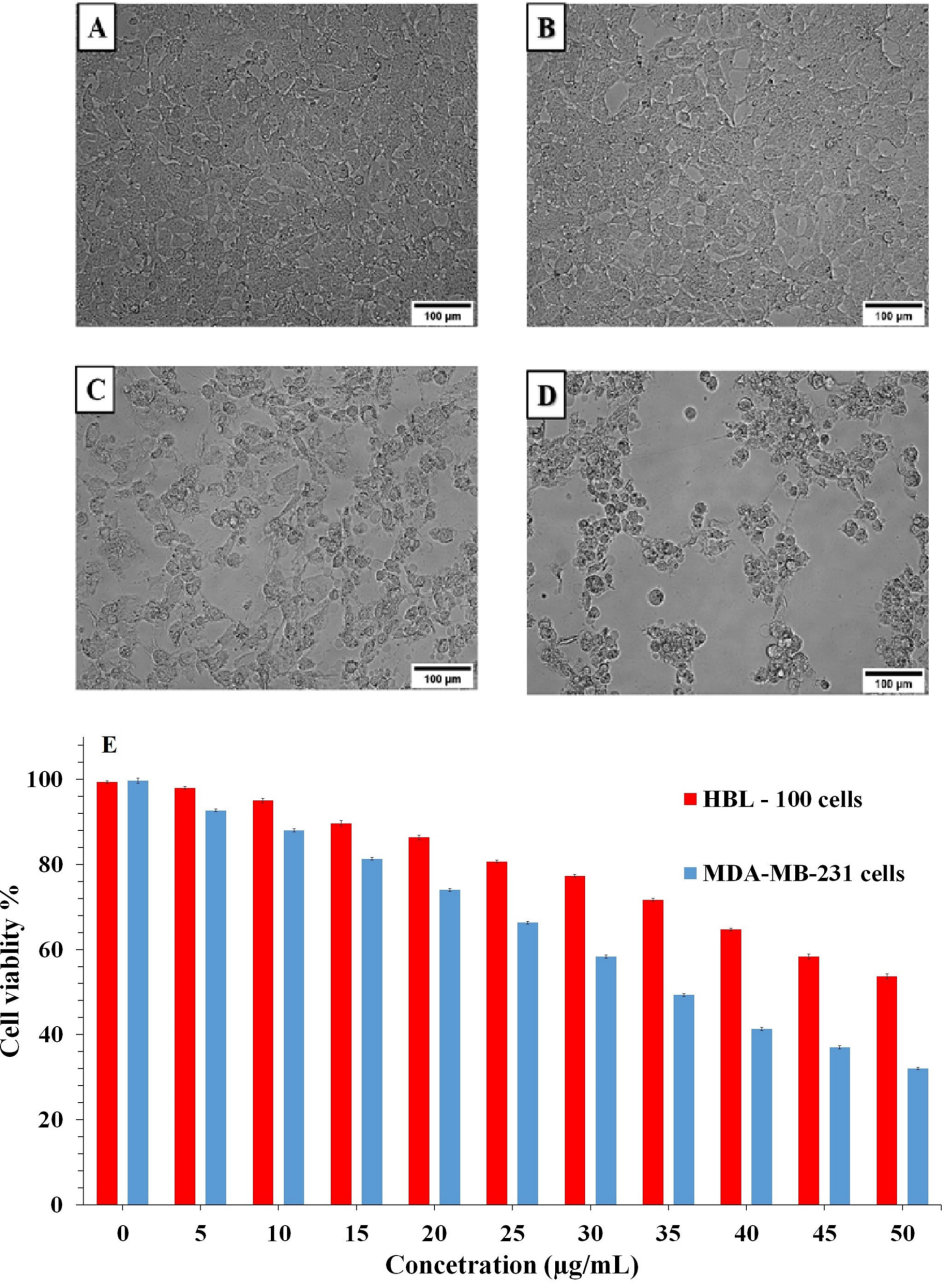
(**A–E**) The MTT assay for relative cell viabilities of the MDA-MB-231 breast cancer cells and HBL-100 normal breast cells incubated with rapid synthesized SeNPs for 48 h. Microscopic images of with and without SeNPs treated cells [(**A**,**C**) HBL-100 normal cell; (**B**,**D**) MDA-MB-231 cell].

Figure 6A–E displays the in-vitro cytotoxic potentiality of SeNPs at different concentrations (0, 5, 10, 15, 20, 25, 30, 35, 40, 45, 50 µg/mL) and its effects on proliferation of HBL-100 cells (Fig. 6A,C) and MDA-MB-231 cells (Fig. 6B,D) using MTT assay. These findings suggest the apoptosis induction could be the possible mechanism for the rapid synthesis of the SeNPs anti-proliferative activity. About 50% of cell death, which determines the rapid synthesized SeNPs inhibitory concentration (IC_50_) value against MDA-MB-231 cells was found to be 34 μg mL^−1^ at 48 h. However, rapidly synthesized SeNPs cytotoxicity assay against HBL-100 cells did not show substantial cytotoxicity at lower concentration and cytotoxicity rises when the inhibitory concentration rise above 50 μg mL^−1^ at 48 h (Fig. 6E, Supplementary Table 1) provides conclusive evidence on the cytotoxic impact of rapidly synthesized SeNPs on the MDA-MB-231 cell line of breast cancer when compared to the normal HBL-100 breast cell line cells^21^.

### In-silico analysis for carrierness of SeNP in Breast cancer protein

**Figure 7:**
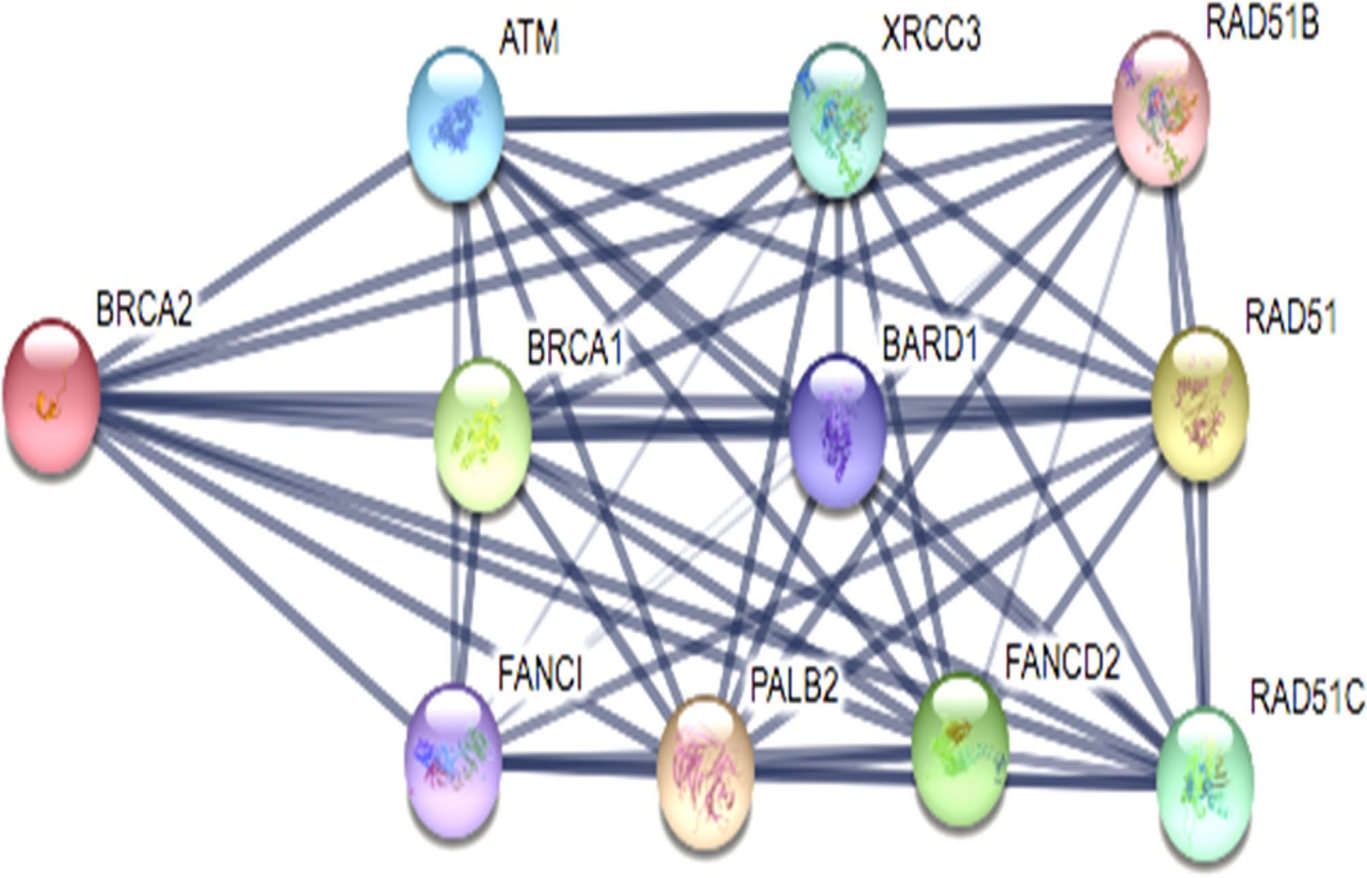
Protein–protein interaction study for the selection of BRCA2 for SeNPs complexity.

**Figure 8:**
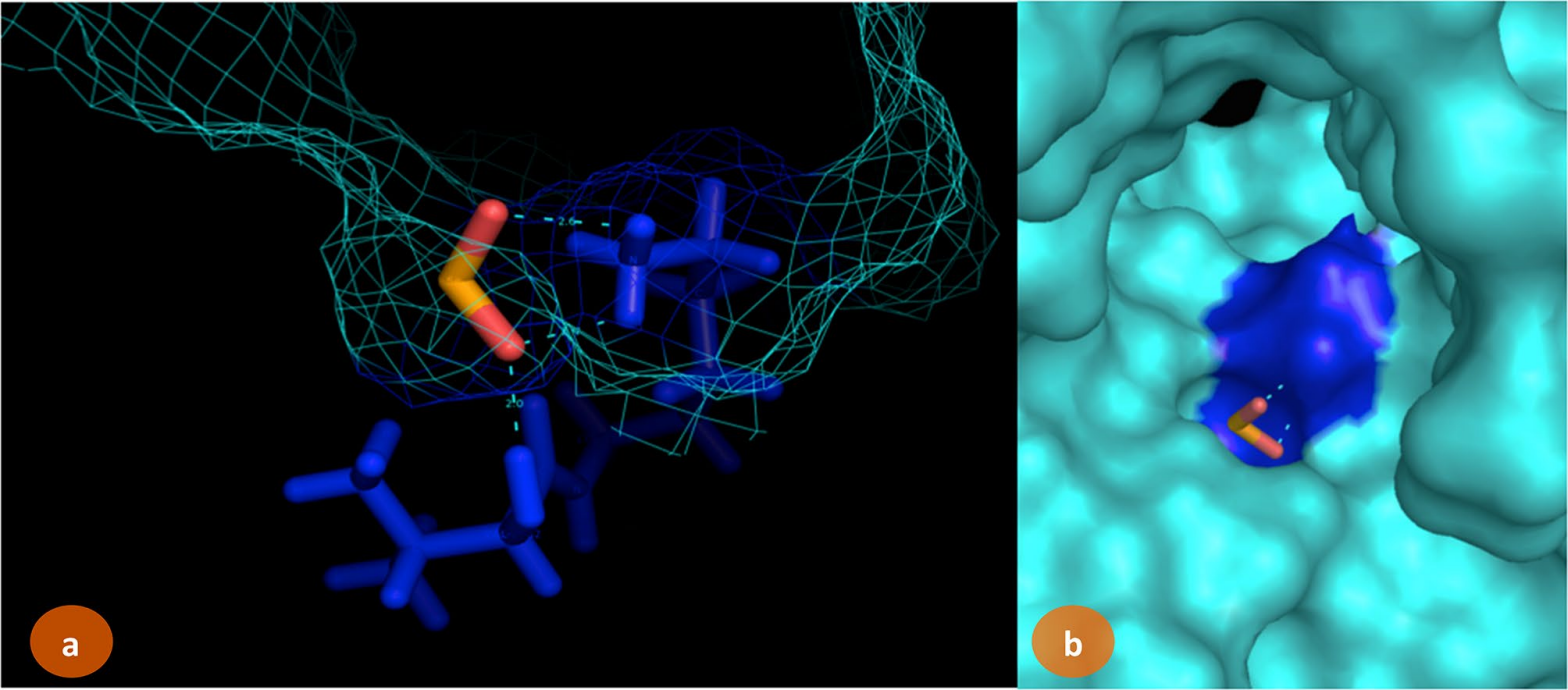
SeO2 interaction map with BRCA2 protein. LYS163 and VAL162 residues involved to interact with SeNPs. Blue colored regions are interacted surface. (**a**) Wired representation of cavity involved to form between NP and residues. (**b**) Surface representation of active site cavity for SeNPs interactions.

**Figure 9:**
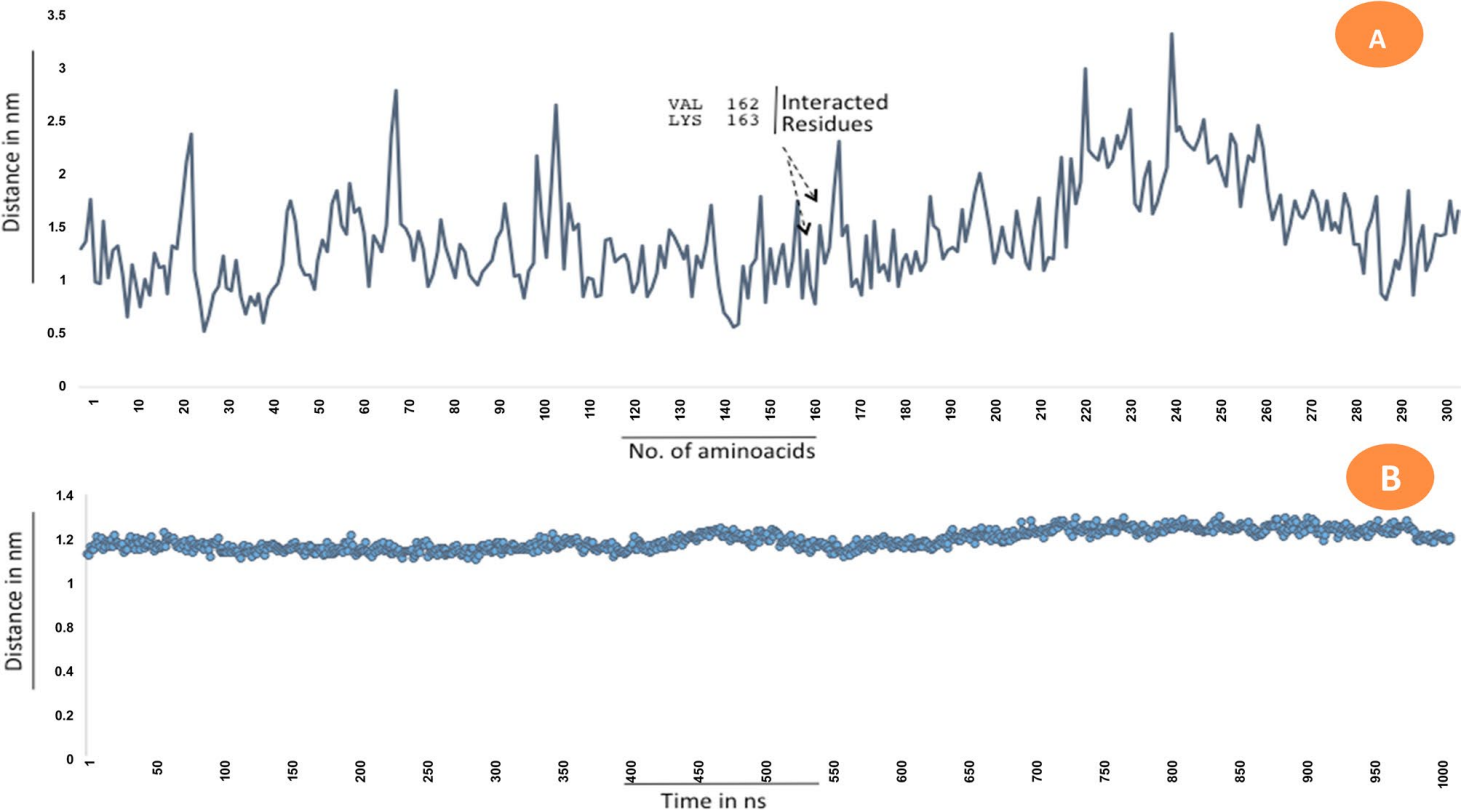
Root mean square deviation of interacted complex and fluctuation dynamics of interacted residues. (**A**) Calcaution plot for residual fluctuation in the system environment for 100 ns. (**B**) rms D calculated for docked complex—100 ns observation.

Selection of breast cancer protein was screened through Protein–Protein interaction network obtained from STRING Database. The centrality and divergence of BRAC2 exhibit equal distributions with the neighbor proteins involving in the inhibition/activation metabolism (Fig. 7). Significantly, BRCA1 showed less threshold frequency among other genes such ATM, XRCC3, RAD51B, BARD1, RAD51, FANCI, PALB2, FANCD2, RAD51C. This hierarchically indicates BRCA2 contains larger connecting nodes than BRCA1. So, the study was further focused to analyse the structure of BRCA2 from Protein Databank and its interaction with SeO_2_. SeNPs are shown to interact with BRCA2 target protein. The BRCA2 protein consists of high electronegative aminoacids on its surface. Complex of SeNPs and BRCA2 protein was calculated for binding energy of about − 6.45 kJ/mol from Glide docking protocol. About 4A3 volume of the cavity computed for SeNPs interacted site. Geometry of SeO_2_ observed static at conformational projections. Selenium placed at 67° angle projection between di-oxide dimension. So the planarity of the SeO_2_ remains same at 360° rotation (Fig. 8). Docking calculation was performed for 100 best pose with multiple planar exposure. The study found that Valine and Lysine interacts at the top interacted poses. Docking geometry of SeNPs emphasize the cavity is involved in interacting with other smaller functional pocket predicted during the course of search algorithm internally. Post-docked complex were subjected to undergo distance and fluctuation calculation to understand the behavior of the SeNPs within its active site cavity and interaction performance with residues involved to form hydrogen bond. Root mean square deviation of interacted complex was observed for 100 ns in the marine environment (Fig. 9). We observed consistent behavior in SeNP suggesting them to be a stable residual activity throughout the simulation period.

### Antibacterial activity of SeNPs

**Figure 10:**
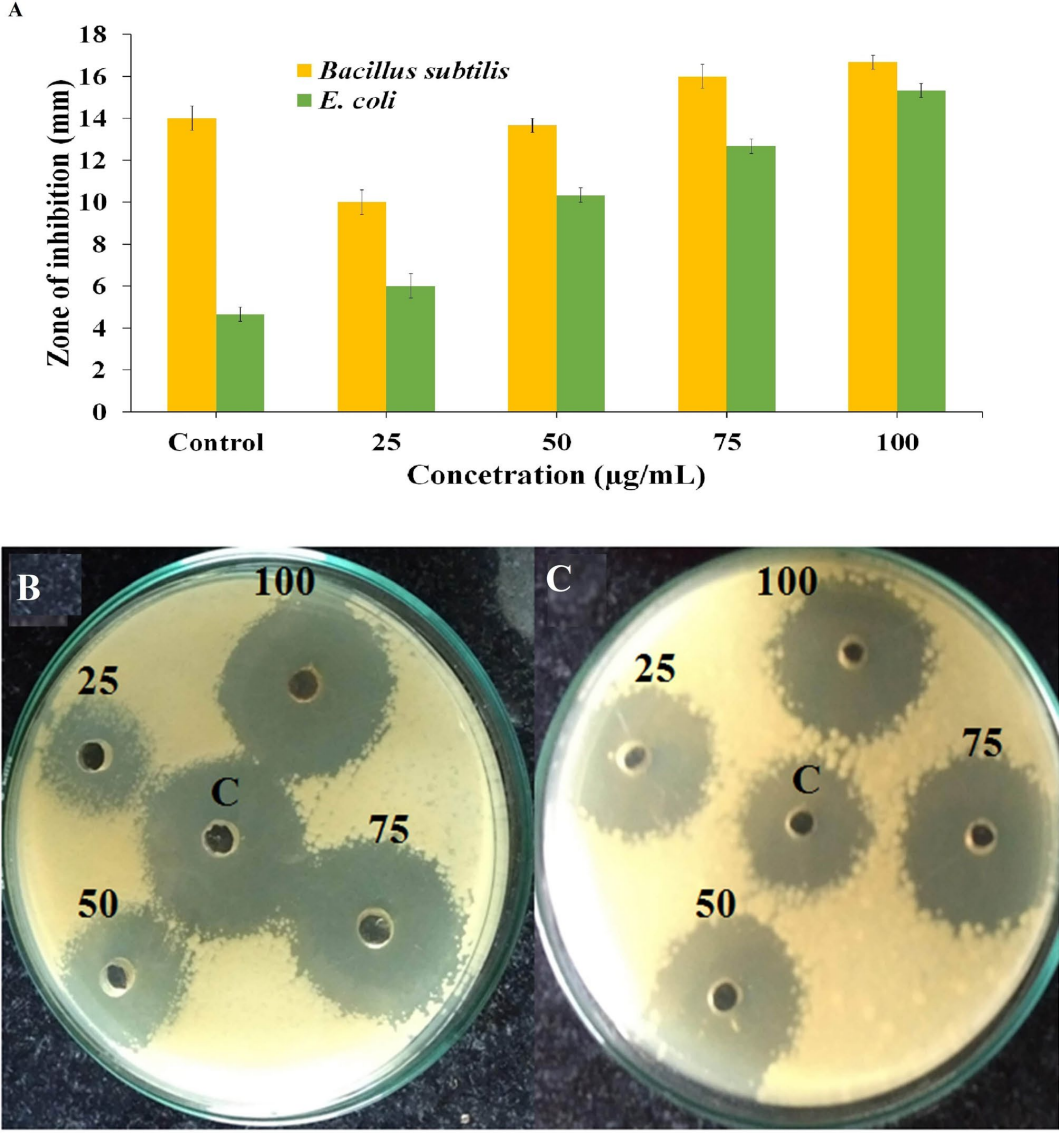
(**A**) The antibacterial activity of SeNPs on different pathogenic bacteria (*B. subtilis* and *E.coli*) at different concentration (25, 50, 75, and 100 µg) and control antibiotic methicillin (The zone of inhibition values are expressed as mean ± SD and analyzed by one-way variance (ANOVA). Representative images of inhibition zones against clinical pathogens, (**B**) *B. subtilis* and (**C**) *E. coli.*

The synthesized SeNPs were tested for its antibacterial activity against clinical pathogens (gram-positive and gram-negative bacteria) at different concentrations like 25, 50, 75 and 100 μL/mL, and a clear inhibition range was seen between 14 and 20 mm diameter (Fig. 10A–C, Supplementary Table 2). Table 1 shows the inhibition results of each concertation and the result was compared with the normal antibiotic methicillin (standard). Overall, these results reveal implication of synthesized SeNPs in pharmaceutical industries. Similar results were obtained from the SeNPs obtained from the leaf extract of *Ficusben ghalensis*, comparable inhibition zones were seen in higher zone of inhibition (ZOI)^22^. The variations might be correlated with the varied antimicrobial activity assessment approaches. Other study showed the efficacy of 50 mL extract of dried *Vitis vinifera* (Raisin) demonstrated comparative outcomes against explicit clinically pathogenic bacterias. Ethanol extract from the branches of *Vitis vinifera* inhibits the *Staphylococcus aureus* (zone of 10–18 mm in diameter) but not the other bacteria’s like *Escherichia coli, Bacillus subtilis* and *Streptococcus faecalis*. Our outcome is in affirmation with this circumstance. Furthermore, microbe’s flexible sensitivity towards chemical molecules is related with the varied resistance levels amongst the strains^17^. Senthilkumar et al. discovered the role of *Laurusnobilis* plant extricated SeNPs as an effective inhibitory agent for *Mycobacterium smegmatus* with the ZOI of 15 mm, this extracted SeNPs is shown to hinder *Listeria monocytogenes*, *B. megaterium* and *S. aureus* growth. The discoveries of this investigation are similar to those previously expressed^23^.

**Table 1:** Rapid synthesized SeNPs antibacterial activity against clinical pathogens.

Pathogenic bacteria	Zone of inhibition in (mm) concentration of SeNPs (µg/mL)				
	25 µL	50 µL	75 µL	100 µL	Control
*Bacillus subtilis*	10 ± 0.81	14 ± 0.47	16 ± 1.24	17 ± 0.47	14 ± 0.81
*Escherichia coli*	6 ± 0.81	10 ± 0.81	13 ± 0.94	15 ± 1.41	5 ± 0.81

**Figure 11:**
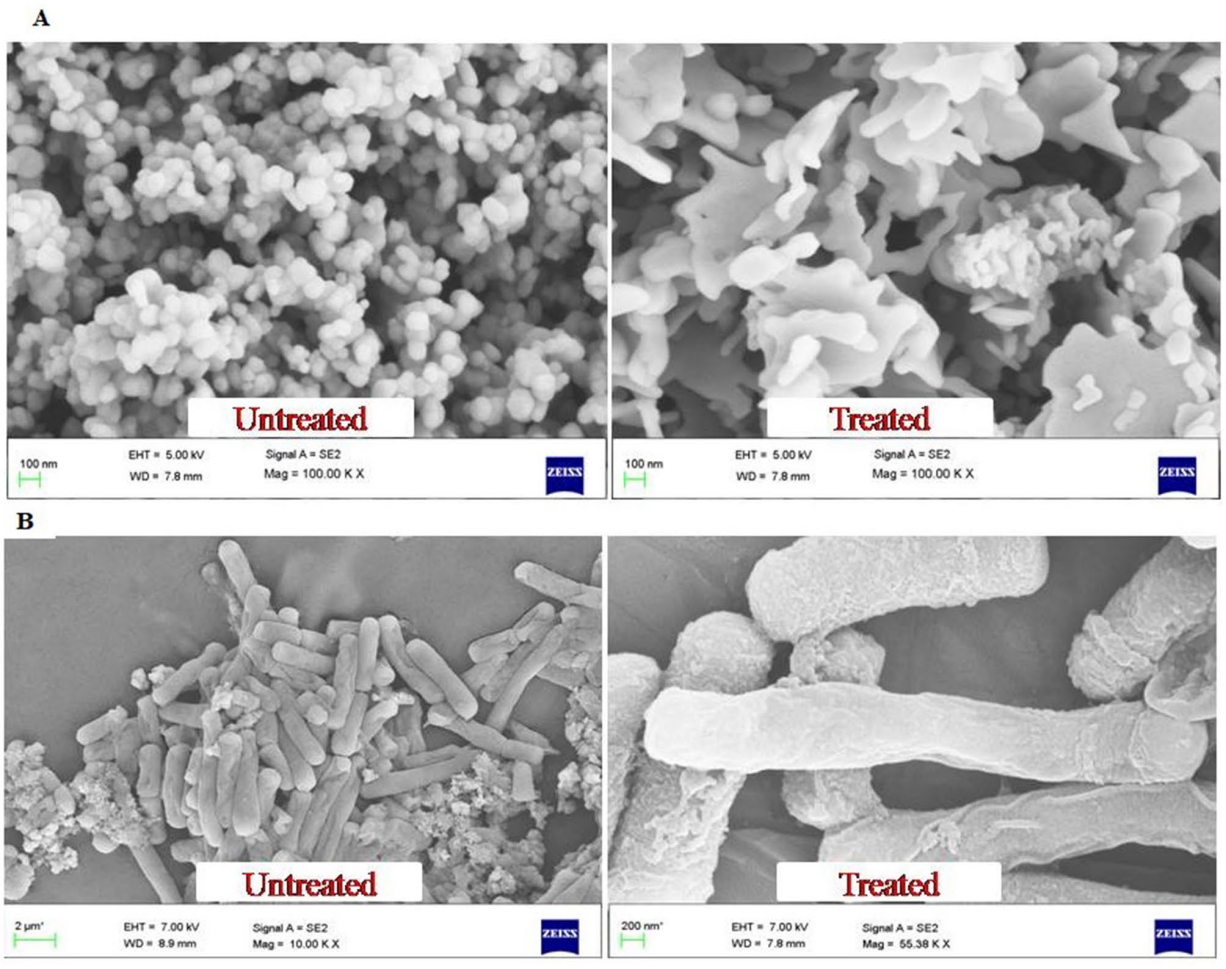
FE-SEM image of rapid synthesized SeNPs treated and untreated pathogenic bacteria in (**A**) *B. subtilis* and (**B**) *E. coli*.

**Figure 12:**
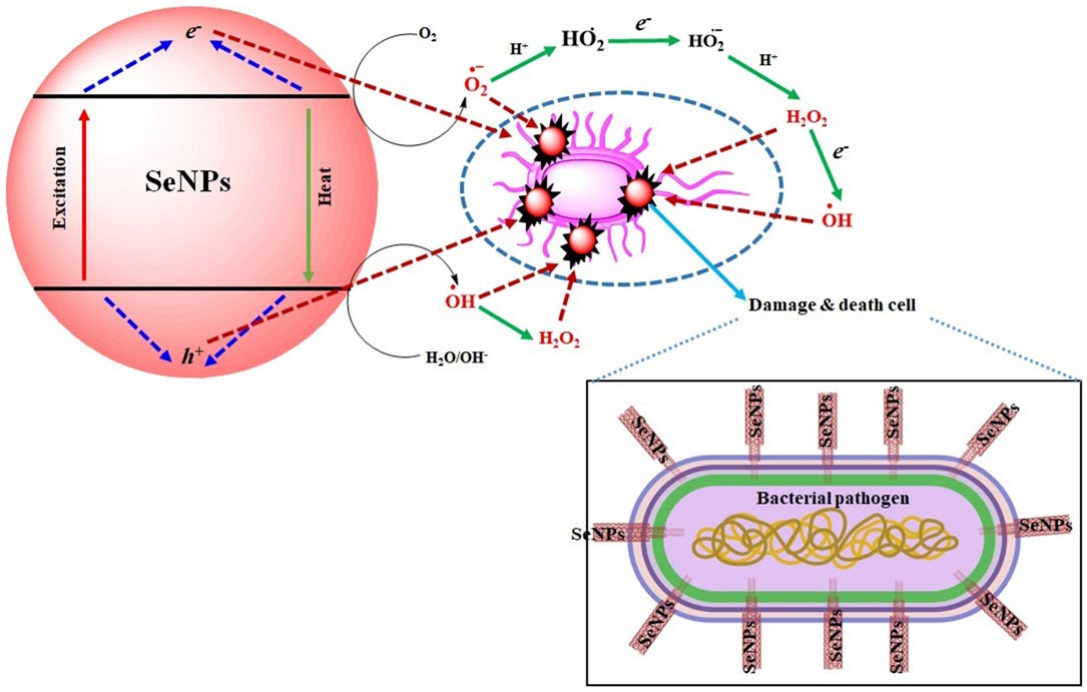
Schematic representation for mechanism of bacterial cell damage by SeNPs.

The ionic interaction between the negatively charged SeNPs and the penetrated gram positive *B. subtilis* or gram negative *E. coli* leads the SeNPs to cause cell damage by destrupting the cell wall^23,24^. The morphological changes of untreated and SeNPs treated *B. Subtilis* and *E. coli* were observed with FE-SEM analysis (Fig. 11A,B). Untreated *E. coli* displays no significant morphological changes were as 50 µg SeNPs treated bacteria displayed significant morphological changes against *B. subtilis*. This may be due to the direct communication of nanoparticles on the membrane surface of *E. coli* cell causes interruption in the cell membrane and inhibit their growth by either blocking cell wall synthesis or inactivating other cellular processes. Figure 12 shows the schematic diagram of SeNPs mode of action in damaging the bacterial cell damage.

### Larvicidal activity

**Figure 13:**
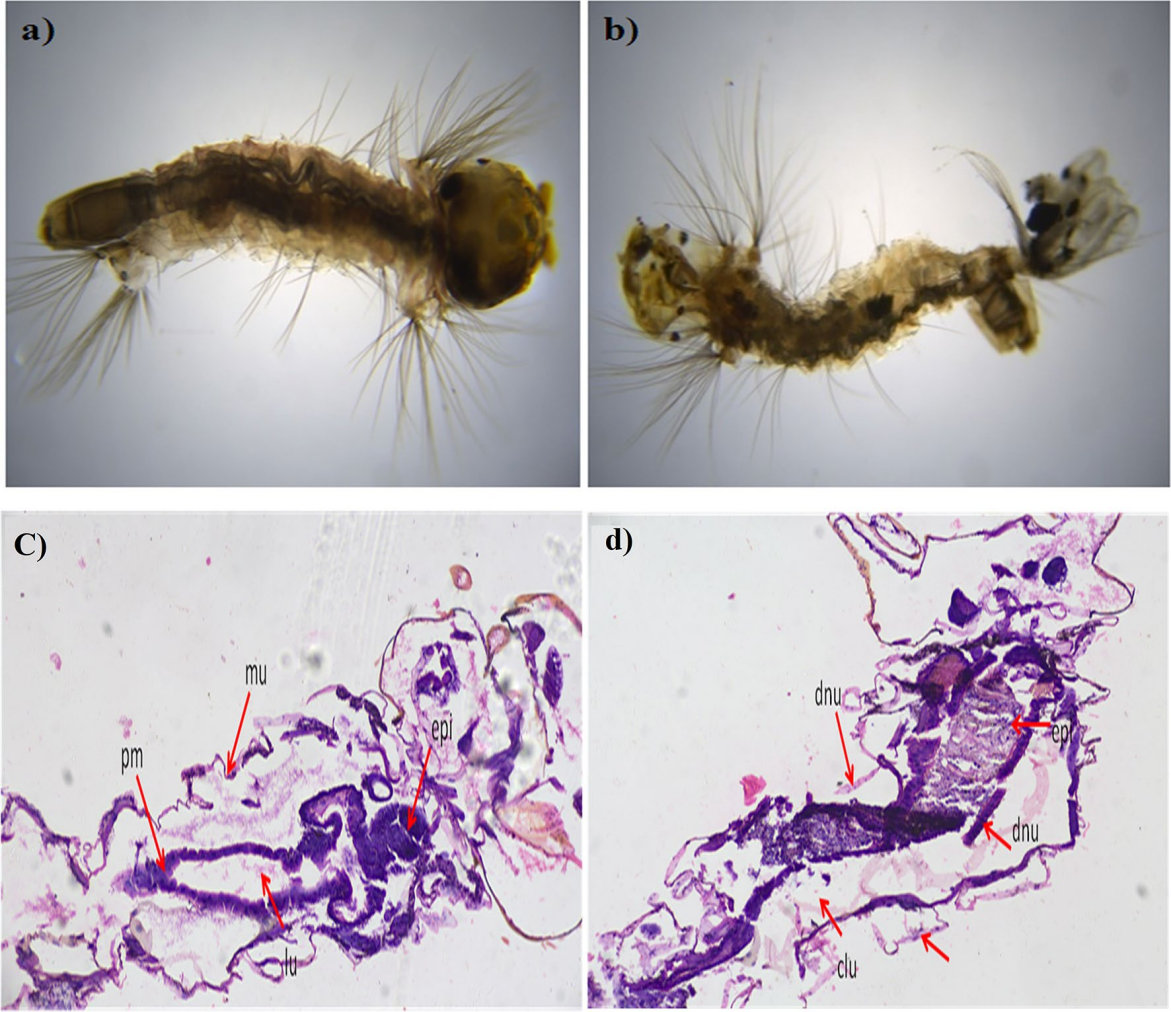
Larvicidal activity of *Ae. albopictus* fourth instar larvae (**a**) Control, (**b**) treated. Histopathological studies of *Ae. albopictus* 4th instar larvae (**c**) Control, (**d**) Rapid synthesized SeNPs treated larvae showing severely damaged and vacuolated gut epithelium (epi) and muscles (mu), damaged gastric (d) caeca (c), gut lumen (lu), peritrophic matrix (pm) at 200 × magnification.

Rapidly synthesized SeNPs showed a potential role in larvicidal activity by providing significant mortality rate at the 4th instar larvae stage for the dengue vector, *Ae. albopictus* (Table 2; Fig. 13a,b) at different concentrations like 10, 20, 30, 40, and 50 mg/mL to analyze the perform of the probe. Highest mortality rate was (82%) observed in 1st instar larvae, when treated with 50 mg/mL SeNPs. Whereas the lowest mortality rate was (26%) recorded at 4th instar larvae stage, when treated with 10 mg/mL SeNPs. The median LC_50_ values of SeNPs against the 1st to 4th *Ae. albopictus* larval instars were 15.22, 32.62, 43.53 and 52.33 mg/mL, respectively and the LC_90_ values are 132.763, 130.925, 128.394 and 178.314, respectively. The present result implicates a positive correlation between the mortality rate and dosage level as reported by Sowndarya et al.^25^. They reported the SeNPs of *Castanea dentata* leaf extract has 240.714 mg/L, 104.13 mg/L, and 99.60 mg/L as the median lethal concentration. Additionally, the mortality rate of *Ae. albopictus* larvae is due to SeNPs penetration through the cell membrane and further reacting with the membrane proteins to hamper its function.

**Table 2:** Rapid synthesized SeNPs larvicidal activity against *Ae. Albopictus larve.*

Mosquito species	Larvae stages	Concentrations mg/L	Percentage mortality	LC_50_ (LCL–UCL)	LC_90_ (LCL–UCL)	χ^2^ (*df* = 3)
*Ae. albopictus*	1st	Control	43.00 ± 0.6	15.222 (7.106–26.574)	132.763 (110.099–201.890)	6.732
		10	57.00 ± 2.0			
		20	61.66 ± 0.5			
		30	72.00 ± 1.0			
		40	78.66 ± 0.0			
		50	82.00 ± 3.1			
	2nd	Control	33.33 ± 1.6	32.621 (9.643–66.998)	130.925 (76.549–138.232)	7.539
		10	39.00 ± 1.1			
		20	45.33 ± 1.0			
		30	51.00 ± 1.2			
		40	54.33 ± 1.0			
		50	64.00 ± 1.2			
	3rd	Control	27.00 ± 1.6	43.536 (11.313–43.237)	128.394 (138.585–242.419)	8.418
		10	32.33 ± 1.5			
		20	38.33 ± 2.3			
		30	43.00 ± 1.0			
		40	53.00 ± 0.0			
		50	59.00 ± 1.4			
	4th	Control	26.00 ± 1.6	52.336 (12.213–52.500)	178.314 (138.585–262.419)	9.431
		10	28.33 ± 1.5			
		20	31.33 ± 0.0			
		30	39.00 ± 1.0			
		40	43.00 ± 1.6			
		50	48.00 ± 1.0

### Histopathological study

The damages caused by SeNPs in the extra and intra cellular lining of the *Ae. albopictus* larval instars were analyzed by the histopathological analysis. The histopathological images revealed the disruption in the epithelial cells of the gut region (Fig. 13c,d). The SeNPs treated larval instar’s cellular components such as nucleus, lumen and gut epithelial cells were affected (Fig. 13d) due to the interaction between the cellular molecules leading to the cellular components damages. In particular, the SeNPs reacts with the cellular protein and there by hinders the enzymatic reactions among the larger mammals. This could be a possible toxic reaction in the invertebrates^26^.

### Photocatalytic activity of SeNPs

**Figure 14:**
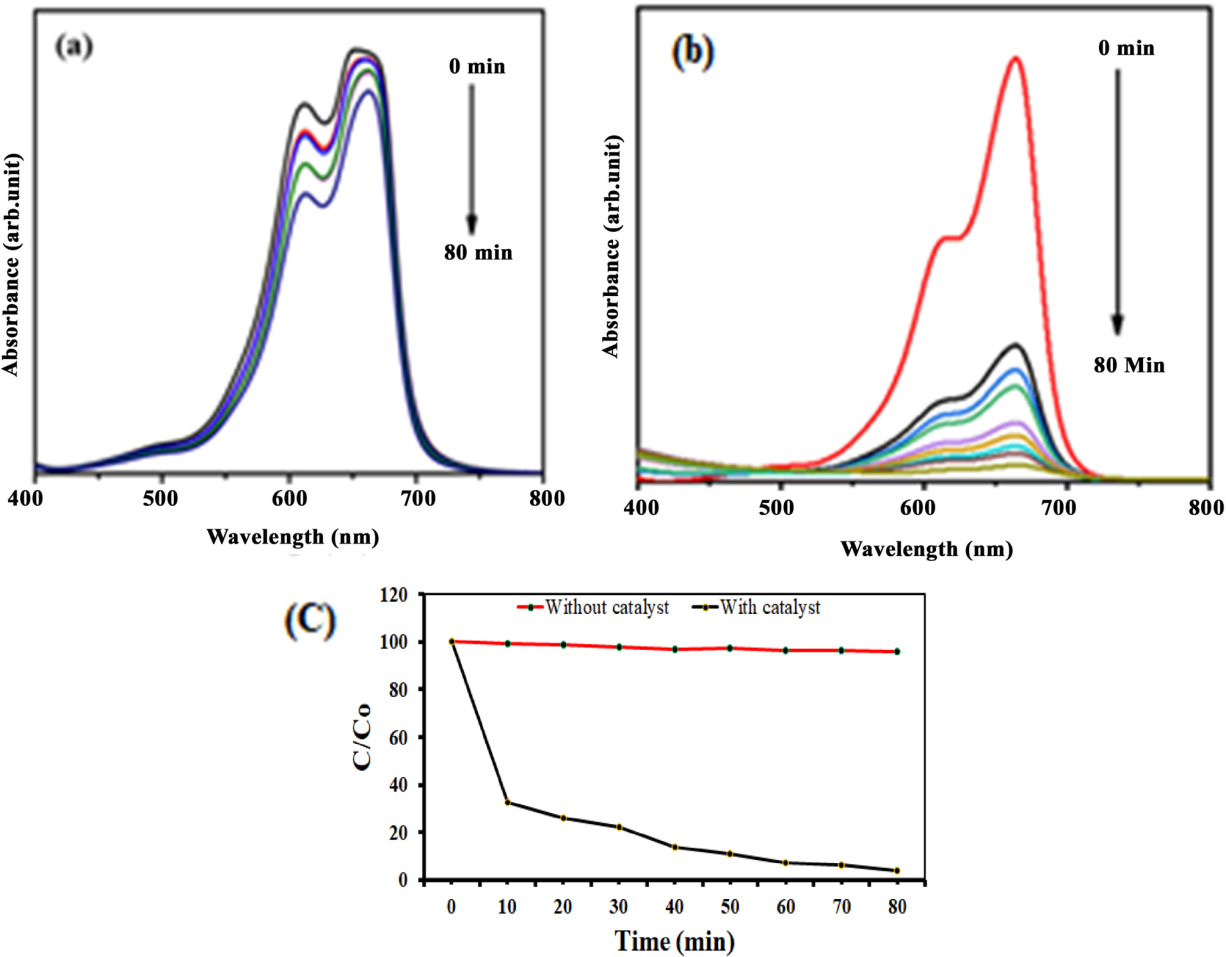
(**a**) Adsorption spectrum of MB under light irritation, (**b**) adsorption spectrum of rapid synthesized SeNPs against MB under light irritation and (**c**) photocatalytic degradation of methylene blue in the presence SeNPs.

**Figure 15:**
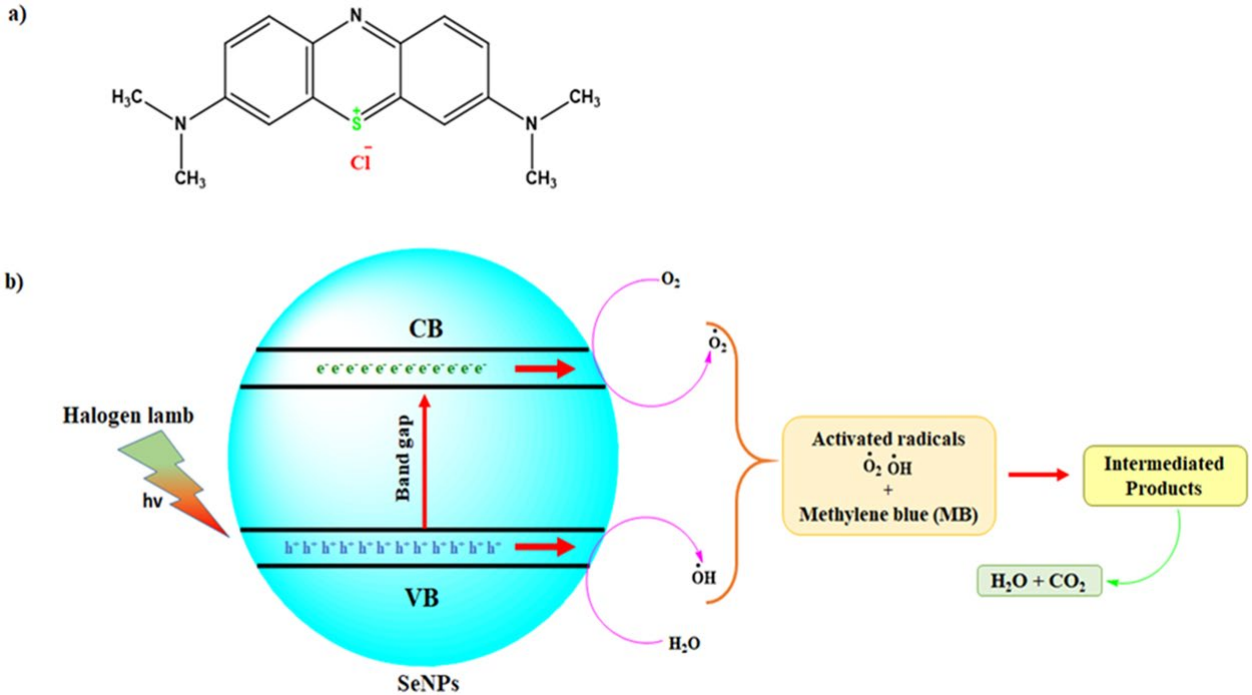
(**a**) Structure of methylene blue (**b**) scheme of the photocatalytic mechanism of the rapid synthesized SeNPs under halogen light irradiation.

Photocatalytic dye degradation of MB was examined by two different analyses namely with and without SeNPs mediated MB dye degradation. The detailed results shown in Fig. 14a–c, the decolonization efficiency of MB was steadily increased with higher irradiation time (up to 80 min). However, compare to the control solution (without SeNPs; Fig. 14a), the SeNPs mediated aqueous solution produced a good degradation rate (98.3%) in 80 min (Fig. 14b). Overall, these free radicals could bleach the MB within 80 min and degrades MB to generate intermediate products (Fig. 14c, Supplementary Table 3). The results evidently suggests the efficacious role of SeNPs in the photocatalysis treatment of the toxic pollutants obtained from textile industries. Figure 15, illustrates the schematic diagram of SeNPs mechanism in the photocatalytic degradation activity of MB dye, the catalyst of SeNPs photo-generated holes in VB (valence band) h_vb_^+^ by reacting with H_2_O to produce OH^-^ and H^+^ species. Further, e^-^ in CB (conduction band) produces an ERP (Electron Resonance Plasma) over the SeNPs surface and they react with O_2_ to produce O_2_^−^^27,28^. The OH—(hydroxyl radicals) and O_2_^−^ (superoxide radical anions) generated enables the oxidation process on the surface of SeNPs catalyst and enrich the MB degradation.

## Conclusion

In this study, SeNPs was rapidly synthesized from *C. bulbosa* tuber aqueous extract and was characterized by UV–Vis spectrophotometer, FT-IR, XRD, FE-SEM-EDS mapping, HR-TEM, ZP and DLS analysis. Rapidly synthesized SeNPs demonstrated significant cytotoxicity in MDA-MB-231 breast cancer cells and HBL-100 normal breast cell at IC_50_ values of 34 µg/mL and 50 µg/mL at 48 h treatment, respectively. The SeNPs showed efficient antibacterial activities against *B. subtilis* and *E. coli*. Molecular interaction between SeNPs and BRCA2 protein showed good interaction access to exhibit the functions of nanoparticles conveyor in the breast cancer target proteins. It also exhibited larvicidal activity in *Ae. albopictus* against the dengue vector. Additionally, MB was effectively degraded under halogen light illumination in the presence of SeNPs. The benefits of using plant tuber extract for synthesis of SeNPs was cost effective, energy efficient, environment and human health friendly plus is the safest product. This ecofriendly method could be used as an potential biomedical and environmental mode of application in the near future.

## Materials and methods

### Materials and reagents

Selenous acid (H_2_SeO_3_), MTT 3-(4,5-dimethythiazol-2-yl)-2,5-diphenyltetrazolium bromide, DAPI 40-6-diamidino-2-phenylindole, acridine orange/ethidium bromide (Ao/EtBr), Sodium dodecyl sulfate (SDS) were purchased from Sigma Aldrich, India. All additional analytical grade chemicals were obtained from commercial suppliers.

### Collection of plant and obtaining the extract

Collecting and identifying the plant species by standard practice and preserving them in the herbarium (Voucher No. BS1/SRC/5/23/2017/Tech/1952) at Botanical Survey of India, Coimbatore. Aqueous extraction from the plants were carried out as per previous report^7^.

### Synthesis of SeNPs from plant extract

In a typical mix of reactions, 5 mL of *C. bulbosa* tuber extract is diluted with 45 mL of double distilled water (DDW), followed with the addition of 20 mL of 40 mM Selenous acid solution. The mixture solution was stirred for 24 h at 37 °C (room temperature) until the color changes from yellow to ruby red. In the end the resultant product is washed with DDW by centrifugation at 10,000 rpm for 10 min. The washing step was repeated several times until the impurities were removed. Finally, the red pellet was dried in freeze drier for two days and used for further study.

### Characterization of SeNPs

The rapidly synthesized SeNPs was characterized by UV–Visible spectroscopy (SHIMADZU-1800, India). The phase formation and crystalline nature of the SeNPs was examined by Rigaku XRD at a voltage of 45 kV with Cu-Kα radiation (K = 1.5406 Å). Functional groups were analyzed by FT-IR (PERKIN ELMER SPECTRUM 100 FT-IR Spectrometer). The IR (Infra-Red) spectrum was recorded in middle region wavelength of 4000–400 cm^−1^ at a resolution of 4.0 cm^−1^. A suspension on a Zeta sizer Nano ZS particle analyzer (MALVERN) was used to measure the surface charge of the SeNPs. The surface shape and particles elemental analysis was carried out using FE-SEM with EDS mapping analysis (JEOL 7401 F) and HR-TEM (TECNAI G2 F30) analysis. The DLS and ZP analyzer (MALVERN ZETA sizer nano-ZS90, UK) was utilized to measure the size dimension and surface charge of synthesized SeNPs.

### In silico analysis; selection of target protein and interaction molecule with SeNPs

Protein selection: selection of protein target was carried out by studying the functional interaction of breast cancer protein through STRING Database. The list of the proteins involved highly in breast cancer protein were retrieved from nodes and edges calculated from the large repositories of protein functions. Molecular Interaction: target protein BRAC2 was retrieved from Protein Databank, 3EU7. Chain A was selected to interact with Selenium Dioxide. SeNP and BRAC2 were interacted using Schrodinger Glide. Molecular dynamics of simulation was performed on docked complex for about 100 ns in water solvent environment. Fluctuation and Deviation dynamics of interacted complex was studied by Macromodel module.

### Cell culture maintenance and treatment

The MDA-MB-231 cells were purchased from National Center for Cell Science (NCCS), Pune, India and maintained in DMEM media supplemented with 10% FBS, 1% 2-mM l-glutamine, 100 U/mL penicillin, and streptomycin solution, at 37 °C and 5% CO_2_ incubator with 95% humidity. The media was changed every two days and the cells were passage through trypsinization prior to confluence^21^.

### Tetrazolium based cell viability assay

Briefly, the MDA-MB-231 (5 × 10^3^ cells/well) cells were seeded in a 96-well flat bottom culture plate with different concentrations of SeNPs (0, 10, 15, 20, 25, 30, 35, 40, 45, and 50 μL/mL) for 48 h to determine the cytotoxic effects using the cell viability assay based on 3-(4,5-dimethylthiazol-2-yl)-2,5-diphenyltetrazolium bromide dye reduction assay. Tetrazolium salt is converted by mitochondrial dehydrogenases in live cells to insoluble formazan. The resulting formazan is dissolved, and the absorbance was determined using an ELISA plate spectrophotometer at 550 nm and 630 nm (BioTek Instruments, Winooski, Vermont). The outcomes were given as the mean of three independent experiments. Concentrations of SeNPs displaying cell viability (IC_50_ values) was then (the total number of viable cells in the untreated control relative to cells) calculated^21,29^.

### Antibacterial activity

The antibacterial activity of rapid synthesized SeNPs was assessed against human clinical pathogens, such as *B. subtilis* and *E. coli* were procured from Department of Microbiology, Periyar University, Salem, India. The ZOI is observed as per Kalaimurugan et al.^30^ protocol with a slight alteration. Bacterial strains were maintained in NB (Nutrient Broth) medium; the experiment was instigated on NA (Nutrient Agar) plates and the bacterial strain were inoculated with a volume of 100 µL of bacterial suspension with different concentrations of SeNPs. The control plates were made using a well consisting of *C. bulbosa* plant extract alone and all the NA plates were incubated at 37 °C for 24 h. Plates were examined at the end of the incubation period and the ZOI of diameter was noted as mean values (n = 3) and was expressed in mm (millimeter).

### Morphological analysis

The *B. subtilis* and *E. coli* was inoculated into 100 mL of NB containing 50 µg SeNPs followed by incubation of 24 h and centrifuging at 10,000 rpm for 10 min to obtain pellet, which was washed thrice in 0.2 mM PB (Phosphate Buffer, pH 7). Further the pellet was fixed by adding glutaraldehyde (2.5%) in 0.2 mM PB at 37 °C for 1 h. The samples were dehydrated in series of different concentration of ethanol (25, 50, 75, and 100%) for 10 min each. After drying they are subjected to FE-SEM and HR-TEM analysis to measure their degree of impact^23^.

### Larval toxicity bioassay and histopathology analysis

The mosquito, *Ae. albopictus* larvae have been acquired from Institute of IVCZ (Vector Control Zoonoses), Hosur, India and were kept in plastic trays containing deionized water under maintained laboratory condition. Before experimentation, the larvae were fed with a mixture of dog food and yeast. All the experiments were carried out at 28 ± 2 °C temperature, 70–80% relative humidity and 12 h photoperiod in dual (light and dark) conditions, respectively. The larval toxicity was analyzed as per the WHO standard guidelines (WHO, 1996) with slight modifications. Accordingly, 25 healthy 1st to 4th instar larvae were introduced separately into a 250 mL bioassay container, loaded with 199 mL tap water and SeNPs at various concentrations (10, 20, 30, 40 and 50 mg/mL). Larval tissues from control and treated *Ae*. *albopictus* 4th instar larvae were exposed to SeNPs were sectioned (8 µm thickness) using a rotary microtome for histopathology analysis according to our previous protocol^7^.

The percentage of larval mortality (LM) was calculated after 24 h of treatment using the following Eq. (1).

1 $${\text{LM }}\left( \% \right) \, = {\text{ A}} - {\text{B}}/{\text{A}} \times {1}00$$

where, A is the survival in the untreated control and B is the survival in the treated sample.

### Photocatalytic activity of synthesized SeNPs

To examine the photocatalytic activity, 50 mg of SeNPs (catalyst) was added into a 100 mL of MB solution (30 mg/L). A high-pressure halogen lamp was used as light source. The MB aqueous solution along with loaded SeNPs was stirred for 80 min to confirm the absorption–desorption equilibrium of MB dye molecules on the surface of the catalyst^31^. The photocatalytic efficiency of SeNPs was evaluated as per Kalaimurugan et al.^32^ protocol. The photocatalytic dye degradation efficiency was calculated using the following equation:

2 $${\text{D }}\% \, = \, \left[ {\left( {{\text{A}} - {\text{B}}} \right)/{\text{A}}} \right)] \, \times { 1}00,$$

where, A is the initial concentration of solution, B is the final concentration of MB solution.

### Statistical analysis

All the data were calculated in mean ± SD pattern by performing in triplicates. Antibacterial test results were calculated for probability (p) value, p < 0.05 considered as statistically significant.

## Supplementary Information

Supplementary Information.

## Data Availability

Data that support the findings of this study will be available from corresponding author upon reasonable request.
