# Supplementary material for: Green synthesis of selenium nanoparticles mediated from Ceropegia bulbosa Roxb extract and its cytotoxicity, antimicrobial, mosquitocidal and photocatalytic activities
Source: Sci Rep. 2021 Jan 13;11:1032. doi: 10.1038/s41598-020-80327-9 (PMC7806947; doi:10.1038/s41598-020-80327-9)
Supplement: Supplementary file 1 — Supplementary Information. [file 41598_2020_80327_MOESM1_ESM.pdf]

Green synthesis of selenium nanoparticles mediated from *Ceropegia bulbosa* Roxb extract and its cytotoxicity, antimicrobial, mosquitocidal and photocatalytic activities

Vetrivel Cittrarasu[1], Durairaj Kaliannan[2,3,†], Dharman Kalaimurugan[2], Viji Maluventhen[4], Murugesh Easwaran[5], Wen Chao Liu[6,*], Balamuralikrishnan Balasubramanian[7,†,*], Maruthupandian Arumugam[1,*]

[1]Ethnopharmacology and algal biotechnology division, Department of Botany, School of Life Sciences, Periyar University, Salem-636011, Tamil Nadu India.

[2]Department of Environmental science, School of Life Sciences, Periyar University, Salem-636011, Tamil Nadu, India.

[3]Zoonosis Research Center, Department of Infection Biology, School of medicine, Wonkwang University, Iksan-54538, South Korea.

[4]Department of Botany, Thiagarajar College, Madurai – 625 099, Tamil Nadu, India.

[5]Nutritional Improvement of Crops, International Centre for Genetic Engineering and Biotechnology, New Delhi 110067, India.

[6]Department of Animal Science, College of Agriculture, Guangdong Ocean University, Zhanjiang, Guangdong 524088, P. R. China.

[7]Department of Food science and Biotechnology, College of life sciences, Sejong University, Seoul-05006, South Korea.

[†]Equally contributed as first author

***Corresponding authors**: maruthumdu82@gmail.com (M.A); geneticsmurali@gmail.com, bala.m.k@sejong.ac.kr (B.B), liuwc@gdou.edu.cn (W.L)

Supplementary Table 1: Raw data for cell viabilities of the MDA-MB-231 breast cancer cells and HBL-100 normal breast cells incubated with rapid synthesized SeNPs for 48 h.

Concertation (µg/mL)	Cell viability %			
	HBL - 100 cell	SD	MDA-MB 231 cell	SD
**0**	99.33	0.3333	99.67	0.577
**5**	98.00	0.3333	92.67	0.333
**10**	95.00	0.5774	88.00	0.333
**15**	89.67	0.6667	81.33	0.333
**20**	86.33	0.5774	74.00	0.333
**25**	80.67	0.3333	66.33	0.333
**30**	77.33	0.3333	58.33	0.333
**35**	71.67	0.3333	49.33	0.333
**40**	64.67	0.3333	41.33	0.333
**45**	58.33	0.5774	37.00	0.333
**50**	53.67	0.5774	32.00	0.284

Supplementary Table 2: Raw data for the antibacterial activity of SeNPs on different *B. subtilis* and *E.coli*

	Zone of Inhibition (mm)			
**Concetration (µg/mL)**	***Bacillus subtilis***	**SD**	**E. coli**	**SD**
**Control**	14	0.5774	4.666666667	0.3333
**25**	10	0.5774	6	0.5774
**50**	13.66666667	0.3333	10.33333333	0.3333
**75**	16	0.5774	12.66666667	0.3333
**100**	16.66666667	0.3333	15.33333333	0.3333

Supplementary Table 3: Raw data of photocatalytic degradation of methylene blue in the presence SeNPs.

Time	Without catalyst	With catalyst
0	100	100
10	99	32.5
20	98.7	26
30	98	22.5
40	97	14
50	97.5	11
60	96.34	7.5
70	96.5	6.5
80	96	4

Figure S1: Average particle size distribution by Image J analysis.

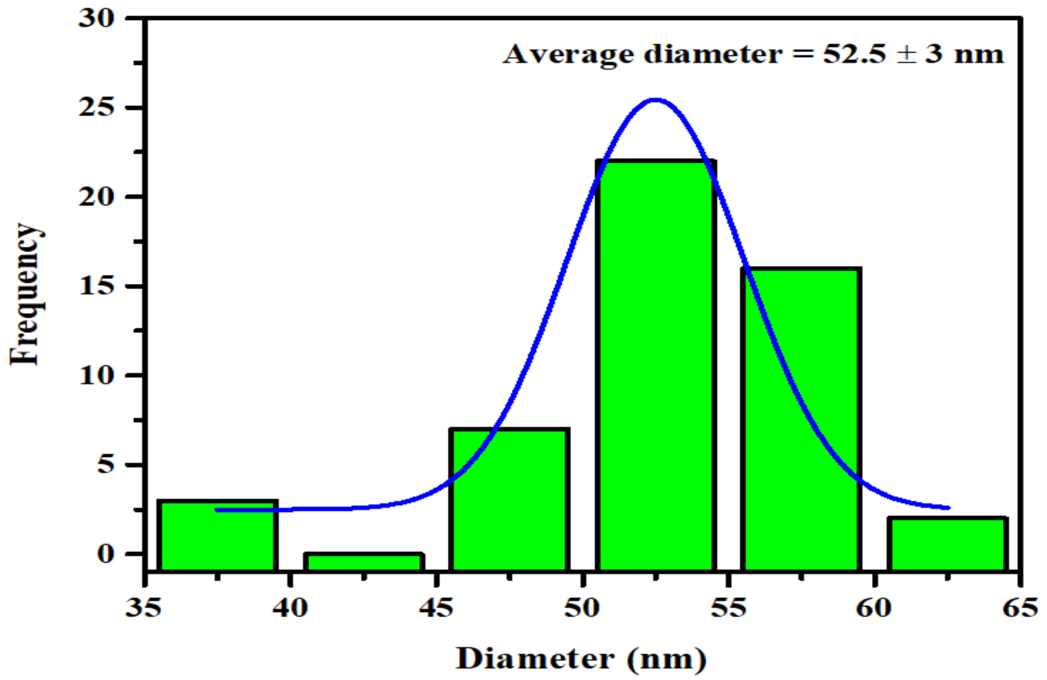

Supplementary Table 4: Histogram raw data for average particle size analysis.

Bins			
**Bin Centers**	**Counts**	**Cumulative Sum**	**Cumulative Probability**
65	0	0	0
75	3	3	6
85	1	4	8
95	7	11	22
105	22	33	66
115	15	48	96
125	2	50	100
